# Fabrication and Computational Study of pH-Responsive Chitosan/Poly(HEMA-co-2-HMBA) Microparticles for Controlled, Site-Specific Doxorubicin Delivery

**DOI:** 10.3390/ijms262110460

**Published:** 2025-10-28

**Authors:** Sivagangi Reddy Nagella, Ramesh Kumar Chitumalla, Jiun Choi, Joonkyung Jang, Hyung Il Seo, Ildoo Chung

**Affiliations:** 1 Department of Polymer Science and Engineering, Pusan National University, Busan 46241, Republic of Korea; 2 Department of Nanoenergy Engineering, Pusan National University, Busan 46241, Republic of Korea; 3 Department of Surgery, Biomedical Research Institute, Pusan National University Hospital, Pusan National University School of Medicine, Yangsan 50621, Republic of Korea; 4 Research Institute of Industrial Technology, Pusan National University, Busan 46241, Republic of Korea

**Keywords:** DOX, chitosan, pH-responsive delivery, controlled release, site-specific release, computational study

## Abstract

As a chitosan (CTS)-based drug carrier (DC) for doxorubicin (DOX) delivery, poly(2-hydroxyethyl methacrylate-co-2-hydroxy-4-N-methacrylamidobenzoic acid) [poly(HEMA-co-2-HMBA)] (PHCH) was successfully grafted onto chitosan to fabricate DOX-loaded microparticles, and their in vitro release behavior was systematicaly investigated. Morphological characteristics were analyzed using scanning electron microscopy (SEM) and transmission electron microscopy (TEM), while DOX loading was validated through Fourier-transform infrared (FTIR) spectroscopy and thermogravimetric analysis (TGA), comparing pure and drug-loaded microparticles. The maximum loading capacity (~91%) was attributed to the presence of abundant carboxylic acid groups, which imparted pH responsiveness during in vitro DOX release. Furthermore, density functional theory (DFT) calculations revealed that hydrogen bonding interactions between DOX and the functional groups of the microparticles strongly influenced encapsulation efficiency (EE%), drug loading (DL%), and release behavior. The fabricated microparticles exhibited pH-dependent DOX release, with accelerated and more complete release at tumor microenvironment pH 5.5 compared to physiological pH 7.4. These results demonstrate that PHCH grafted CTS microparticles are promising candidates for controlled and site-specific anticancer drug delivery.

## 1. Introduction

Cancer is a complex genetic disease caused by specific changes in genes that regulate cell proliferation and homeostasis. Thus, cancer cells undergo unregulated growth, forming a mass or lump of tissue. Furthermore, these cells have the potential to spread to other locations in the body, where they can grow in an irregular manner to produce a malignant growth. Because chemotherapy is one of the best methods to eradicate cancerous tissues [1,2,3], efficient drug delivery systems (DDSs) should be developed to increase the bioavailability of drug molecules in target tissues.

Drug delivery efficiency is an important parameter in the treatment of diseases [4,5,6]. Therefore, the development of a high-efficiency DDS is a challenging task, as most pharmaceuticals face issues such as lower adsorption due to large molecular weight, slow onset of action due to systemic drug administration, and non-selectivity of drug delivery, leading to potential side effects in off-target tissues [7,8,9,10,11]. Nevertheless, the non-selectivity of these anti-cancer agents causes unintended damage to healthy normal tissues, resulting in undesirable side effects [12,13,14]. In addition, their poor bioavailability often requires higher dosages, increasing toxicity in normal cells. To overcome these limitations, the development of targeted chemotherapeutics through either passive or active mechanisms is essential to enhance efficacy while minimizing side effects. Among these, stimuli-responsive targeting systems offer a powerful platform for precision oncology. Stimuli-responsive targeting systems constitute a sophisticated class of drug delivery platforms distinguished by their exceptional precision, tunability, and therapeutic efficacy compared with conventional passive and active targeting strategies [15,16,17,18,19]. They are engineered to dynamically respond to specific endogenous stimuli such as pH, redox gradients, enzymatic activity, or reactive oxygen species and exogenous triggers like magnetic fields, light irradiation, heat, or ultrasound [20,21,22]. Therefore, smart stimuli-responsive systems enable precise spatial and progressive control over drug release within the tumor microenvironment, thereby optimizing therapeutic efficacy while significantly reducing systemic toxicity [23,24]. Drug delivery systems based on pH-responsive mechanisms are particularly effective for tumor treatment, as tumor tissues exhibit a relatively acidic microenvironment, with pH values ranging from approximately 5.6 to 6.8 [25,26,27,28,29,30,31].

More recently, considerable efforts have been focused on the localized delivery of DOX with macromolecular-based drug carriers, including natural and synthetic polymeric structures (micro/nano particles), polymeric micelles, and liposomes [32,33,34,35]. Among these carriers, carbohydrate-based polymeric structures such as chitosan, pectin, etc., have gained significant interest due to their interesting properties [36,37,38]. Owing to their many attractive properties, such as excellent biocompatibility, biodegradability, non-toxicity and non-immunogenicity, chitosan has been widely used as an effective material in drug delivery systems [37,39]. CTS is second most abundant naturally occurring polymer after cellulose, predominantly found in the outer shells of a wide range of invertebrates and in the cell walls of fungi [40,41]. It is a polycationic heteropolysaccharide composed of β-1,4-linked D-glucosamine and partially N-acetylated D-glucosamine, which is generally obtained by the de-acetylation of chitin, extracted from the shells of crustaceans [41].

Doxorubicin (DOX) is a highly potent, hydrophilic anthracycline-type anticancer agent derived from Streptomyces peucetius, widely used to treat a broad spectrum of cancers [42,43,44]. However, its clinical use is limited by dose-dependent side effects, including severe cardiotoxicity and nephrotoxicity [45,46]. Recently, Banendu et al. developed a dual-targeting pH-responsive nanocarrier composed of superparamagnetic Fe_3_O_4_ and oleoyl-modified chitosan conjugated with folic acid (FA) to encapsulate Fe_3_O_4_ and doxorubicin (DOX) [47]. The nanoparticles showed pH-sensitive behavior, with faster DOX release (71%) at endosomal pH (~5.5) than at physiological pH (45%), and enhanced uptake in U87 glioblastoma cells via folate receptor-mediated endocytosis. In another study, a nanocarrier system was developed for controlled drug release within the tumor microenvironment, enabling preferential release of chemotherapeutic agents under tumor-specific pH and redox conditions [21]. This stimuli-responsive behavior enhanced drug accumulation at the target site and improved intracellular delivery, resulting in superior therapeutic efficacy compared to non-responsive systems. A dual pH- and temperature-responsive carrier composed of a PNIPAM–DOX hydrogel inside pH-sensitive polymersomes enabled controlled DOX release, showing faster release at acidic pH (5.5–6.5) and slower release at 37 °C due to hydrogel gelation [18]. In C26 tumor-bearing mice, this dual-responsive system achieved strong tumor inhibition with reduced systemic toxicity and minimal body-weight loss compared to free DOX. pH-responsive gelatin nanoparticles were developed for controlled doxorubicin (DOX) release, exhibiting significantly higher release (~95%) under acidic conditions (pH 3) compared to physiological pH (24%) [17]. Ultimately, this system offers a smart, predictive platform that combines pH-sensitive carrier design with data-driven modeling, enabling optimized drug release profiles and minimizing experimental complexity.

In this work, chitosan/PHCH DC microparticles were produced by free radical polymerization followed by crosslinking with GA. The microparticles were then encapsulated with DOX and characterized to investigate their drug release properties and the mechanism of release. Furthermore, computational studies were conducted to examine the interactions between DOX and the various functionalities present in the microparticles.

## 2. Results and Discussion

### 2.1. Characterization

#### 2.1.1. Fourier-Transform Infrared (FTIR) Spectroscopy

**Figure 1:**
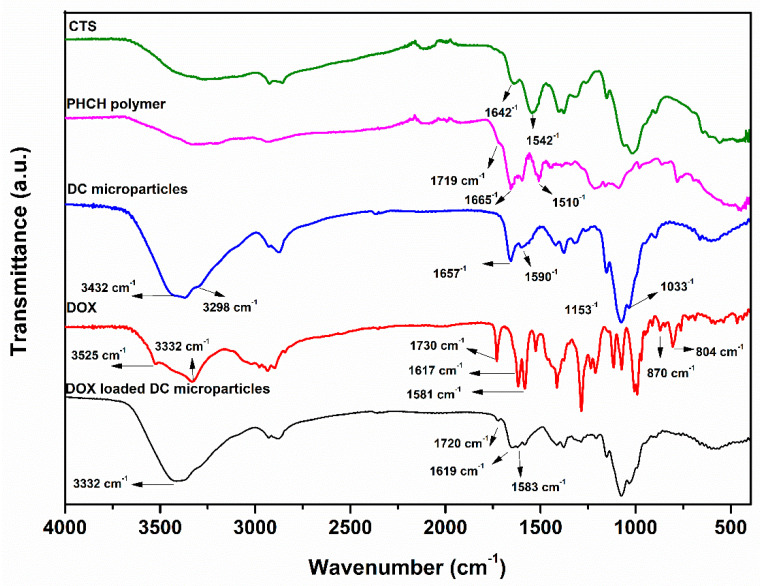
FTIR spectra of CTS, PHCH, DC microparticles, DOX, and DOX-loaded DC microparticles.

The functionality of the DC microparticles and the loading of DOX were characterized by FTIR spectroscopy. The FTIR spectra of DOX, DC microparticles, and DOX-loaded DC microparticles along with CTS and PHCH polymer are presented in Figure 1. The FTIR spectrum of CST exhibits characteristic absorption peaks corresponding to -O-H, -N-H, C-H, and amide functional groups. A broad band in the range of 3200−3500 cm^−1^ is attributed to -O-H and -N-H stretching vibrations, while peaks around 2800–2900 cm^−1^ correspond to C–H stretching. Additionally, distinct bands at approximately 1642 cm^−1^ and 1542 cm^−1^ are assigned to the amide I (C=O stretching) and amide II (-N-H bending) vibrations, respectively. In contrast, the FT-IR spectrum of PHCH displays absorption peaks at 1719, 1665, and 1510 cm^−1^, corresponding to ester, amide I, and amide II groups, respectively. In the case of DC microparticles, a characteristic broad peak is observed at 3371 cm^−1^ along with shoulder peaks at 3432 and 3298 cm^−1^ corresponding to -O-H and -N-H stretching vibrations of chitosan and 2-HMBA. The peaks appearing at 1657 and 1590 cm^−1^ are due to carbonyl of primary amide group stretching and -NH bending vibrations, respectively. The absorption bands at 1153, 1078, and 1033 cm^−1^ are skeletal vibrations involving C-O stretching [39]. In the FTIR spectrum of pure DOX, the characteristic peaks at 3525, 3332, 1730, 1617, 1581, 870, and 804 cm^−1^ are assigned for -N-H, -O-H, C=O, N-H bend and wag, respectively [10]. The FT-IR spectrum of DOX-loaded DC microparticles, shown in Figure 1, shows peculiar peaks at 1720 cm^−1^ (C=O in ester group), 1619, and 1583 cm^−1^ (C=O, -NH bending in amide), which were broadened and shifted to higher frequency, with the concurrent disappearance of -N-H wagging peaks at 870 and 805 cm^−1^ in pure DOX. Thus, the above spectral information confirms the successful fabrications of both microparticles and the loading of DOX into polymer microparticles.

#### 2.1.2. Thermo-Gravimetric Analysis (TGA)

**Figure 2:**
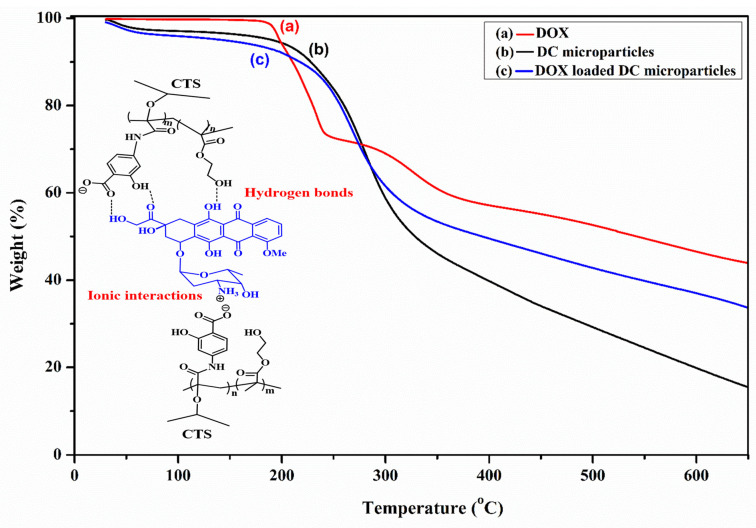
TGA thermograms of (a) DOX, (b) DOX-loaded DC microparticles, and (c) DC microparticles (the inset shows a schematic representation of the interactions between DOX and the PHCH polymer, illustrating both ionic and hydrogen bond interactions).

TGA is an analytical technique used to evaluate the thermal stability of a material by monitoring the weight loss of the sample with temperature changes, when heated at a constant rate in a controlled environment. As presented in Table S1 and Figure 2, three samples, such as DOX.HCl, as prepared DC microparticles and drug-incorporated microparticles were characterized with TGA studies to understand their thermal stabilities and the entrapment of DOX.

The thermogram of DOX.HCl (Figure 2a) displays a characteristic two-stage decomposition curve, and 57.7% of total weight loss was observed in the analysis, ranging from 30~650 °C, which states that the DOX would not completely decompose to volatile gases but remain as a charred mass in the crucible [48]. The first decomposition was observed in the temperature range between 147 and 257 °C, with weight loss of 27.50%, while the second weight stage observed in the temperature range of 257~364 °C accounted for up to 12.79% of loss of DOX weight. The TGA curve of CTS/PHCH (Figure 2b) exhibits a single-stage decomposition beginning at 156 °C and continuing up to 384 °C, corresponding to a weight loss of approximately 54.70%, after which the residual mass stabilizes at 15.45%. In the case of the TGA profile of DOX-incorporated microparticles (Figure 2c), approximately 70% of the material decomposed across the entire temperature range of 30–650 °C. This weight loss is attributed to the reduced thermal degradation of the DOX-loaded microparticles due to the formation of the DOX–PHCH complex. The increased thermal stability of the DC microparticles upon DOX incorporation suggests strong ionic interactions between the NH_3_^+^ ions of DOX and the carboxylate ions of the 2-HMBA side chains in the PHCH backbone, along with additional hydrogen bonding interactions, as illustrated in Figure 2. Similar observations have been previously reported by Manocha et al. [49].

#### 2.1.3. Morphologies

**Figure 3:**
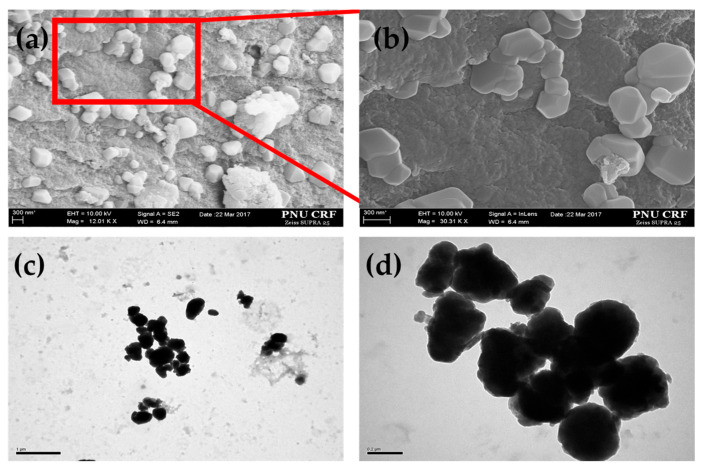
SEM images at different magnifications of DC microparticles (**a**,**b**); TEM images of the same (**c**,**d**).

Figure 3 illustrates the SEM and TEM images of the morphologies of DC microparticles, which shows irregular shapes with average diameters of 300 ± 50 nm. The SEM images of DC microparticles, captured at 12 KX and 30 KX magnifications, reveal irregularly shaped particles with smooth surfaces and variable dispersity due to attachment of particles. The TEM images further support the SEM observations, revealing that the DC microparticles possess irregular yet well-defined shapes with uniform contrast, indicative of a dense internal structure. The particle sizes determined from TEM analysis range between 150 and 400 nm, which aligns with the microscale dimensions desirable for efficient drug carrier systems. These observations confirm the successful fabrication of well-defined and stable DC microparticles via redox-mediated graft polymerization, followed by phase inversion and GA crosslinking.

### 2.2. Drug Entrapment and Drug Release Studies

After loading with free DOX, the DC microparticles were characterized using TGA, FTIR, and drug loading (% DL) prior to the assessment of drug release. The drug encapsulation efficiency (% EE) and DL (%) are the general terms in the drug delivery, which affect the drug release studies directly and are important to maximize the therapeutic efficacy of the drug delivery system. However, the drug entrapment to the polymeric gel and its release depends on several factors, such as (i) the initial concentration of polymer and its functionality, (ii) the ratio between polymer and drug, (iii) the type of interactions between drug and polymer, and (iv) the type of and amount of cross-linker, which controls the swelling ratio of polymer gels. Table 1 shows the encapsulation efficiencies of various DCFs based on CTS microparticles. The EE (%), varying from 30.25 ± 1.6 to 39.91 ± 1.2% depending on the different DCFs, increased with increasing 2-HMBA and cross-linker amount for DCFs A to D and F, C and G, respectively.

Similar results were also found in DL (%), ranging between 54.51 ± 2.5 and 91.77 ± 0.9. These results indicate that the increasing EE (%) is attributed to the increase of polar carboxylic acid groups and cross-linking density in the network. Here, the higher values of EE (%) and DL (%) for DCF-A can be attributed to the higher possibility of hydrogen bonds with DOX because of the highest amount of 2-HMBA, whereas the lowest values for DCF-G, which has the highest amount of cross-linking density, are brought on by the least porosity of the microparticles.

As the microparticles are fabricated with the help of CTS and 2-HMBA, they possess a pH-responsive property, owing to the protonation in response to the slightly acidic medium, which makes the switching of DOX release from the DCF. Thus, the microparticles can show the on-demand release of DOX in tumor tissues due to their relatively lower pH compared to normal tissues. Considering this divergence, the two sets of in vitro release studies were performed for all seven DCFs under two different pH conditions, such as 7.4, a physiological pH, and 5.5, an acidic tumor condition. The required amount of DOX-encapsulated microparticles was dispersed in aqueous buffers and continuously stirred in the shaking incubator. Then, the release medium was subjected to UV-visible spectroscopy at predetermined intervals of time to determine the release of DOX from the microparticles, which was further used for the calculation of cumulative release. Finally, plots were drawn for the cumulative DOX release as a function of time.

**Figure 4:**
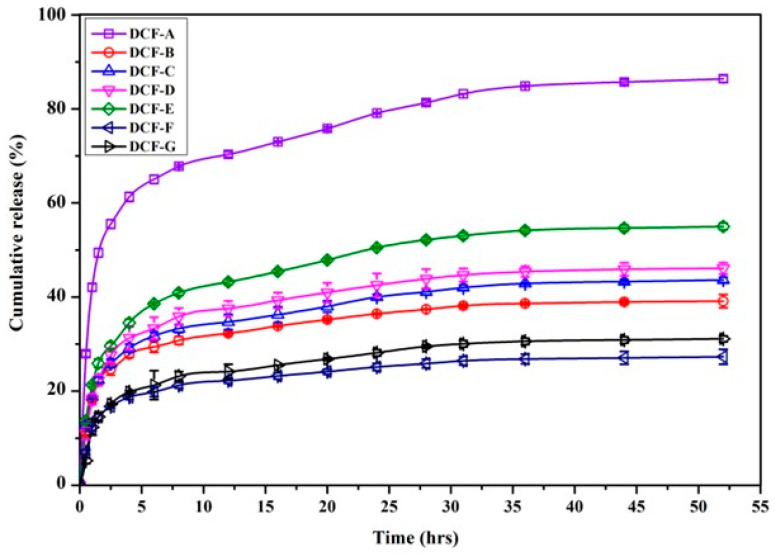
Cumulative DOX release profile of DC microparticles at pH 7.4.

DOX release kinetics of DC microparticles were studied with phosphate buffer saline using a bath method. Figure 4 shows the plot of cumulative DOX release behaviors from microparticles at pH 7.4. The release kinetics of microparticles are different for the all seven DCFs and depend on the amount of 2-HMBA and cross-linker used in the preparation of microparticles. All the DCFs showed an initial burst release of DOX from microparticles in the first hour of the study due to the drug molecules adsorbed on the surface of microparticles, followed by slower release rates, and eventually reaching a maximum cumulative release at 36 h. In the case of DCF-A, a slower and lower drug release was initially anticipated; however, an opposite trend was observed. This unexpected behavior can be attributed to the enhanced interactions between the main polymer chain (CTS) and the graft homopolymer chains of 2-HMBA. The phenolic -OH group of 2-HMBA possesses a low radical propagation rate and mild terminating characteristics, resulting in the formation of shorter polymer chains grafted onto CTS [50]. Consequently, the strong repulsive interactions between the carboxylate ions (-COO^−^) of the 2-HMBA chains promote faster swelling of the polymer network. During this initial polymer relaxation period, the soluble DOX molecules diffuse rapidly via concentration gradient. Over time, the release rate decreases due to stronger electrostatic interactions between the carboxylate ions and DOX molecules, resulting in a slower, sustained release profile. However, as the 2-HMBA content decreases, such repulsive forces are reduced due to the dispersion of negative charges along the copolymer chains.

**Figure 5:**
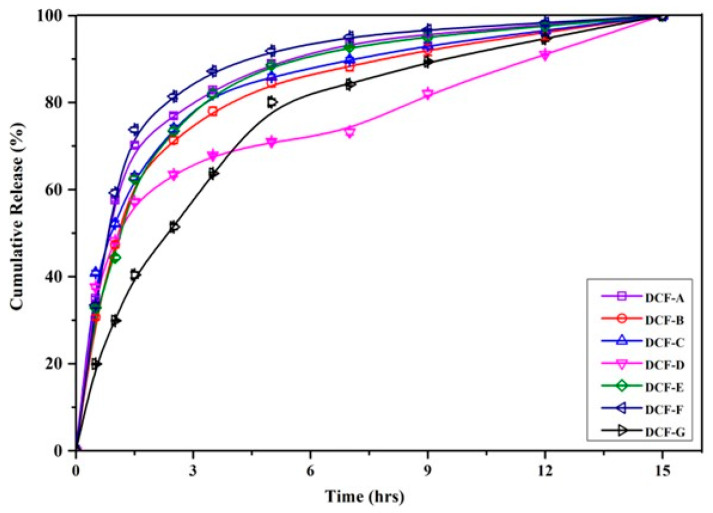
Cumulative DOX release profile of DC microparticles at pH 5.5.

Figure 5 displays the cumulative release of DOX at pH 5.5, showing a higher release rate of DOX from microparticles than at pH 7.4. This effect can be ascribed to both the inherent swelling ability of the polymer matrix and the interactions occurring between the polymer network and drug molecules [47]. However, CTS possesses a pKₐ in the range of 6.3 to 6.5, depending on its degree of deacetylation, and exhibits protonation of its amino groups below this pH, leading to enhanced water uptake by CTS-based materials [51]. Thus, the resulting switchable delivery of DOX was achieved through the pH-responsive properties of CTS. However, the -NH_2_ groups in chitosan are present as neutral molecules at physiological pH and protonated in acidic tumor microenvironments at pH 5.5 to form their ionized -NH_3_^+^. All the DCFs show slower release of DOX at pH 7, which can be attributed to the strong ionic and hydrogen bonding interactions between network groups and drug molecules in comparatively more swollen gels, whereas the faster and higher drug release observed at pH 5.5 may be due to the strong ionic repulsions between -NH_3_^+^ groups of CTS and DOX at a sufficiently close distance in a compact gel network. As a result, the higher drug release was observed at pH 5.5 due to the pronation of amine groups in the CTS and DOX causing the electrostatic repulsion between chains as well as chains and DOX; hence, the swelling and solvent penetration increased. Further, the amount of cross-linking has a bigger effect, since the amount of network swelling largely controls the release of DOX in DC microparticles. The network’s porosity plays a significant role in the diffusion of solvent and medication molecules. As a result, the sequence DCF-F, DCF-C, and DCF-G showed a slower release rate as the cross-linking quantity increased.

### 2.3. Drug Release Modelling

In order to determine the rate and mechanism of DOX release, the data obtained from the DOX release studies were fitted with three empirical kinetic models, such as Higuchi, zero-order, and first order. As presented in Table 2, for both pH conditions, the first order model was considered to be the best one, better than the others due to its higher regression (r^2^) and lowest sum of squares of residuals (SSR) values, which were used to decide the best-fit model. Furthermore, it clearly states that the DOX release behaviors from all DCFs were diffusion controlled.

Semi-empirical models such as the Ritger–Peppas and Peppas–Sahlin models were also applied to confirm the release type of diffusion that plays in the release of DOX from the microparticles. Table 3 reveals that the release of DOX at pH 5.5 was best with the Peppas–Sahlin model due to its highest r^2^ values of 0.9972 to 0.9733 and lowest SSR values of 0.0224 to 0.002, whereas at pH 7.4, the Ritger–Peppas model was best fitted because it had the highest r^2^ values of 0.9779 to 0.9601 and lowest SSR values of 0.021 to 0.0031. However, from the values of ‘n’, it can be known that the release of DOX followed a Fickian type due to the fact that all the ‘n’ values are less than 0.45. The fraction of drug release (F) calculated from Equation (9) indicates that DOX release at pH 7.4 was predominantly diffusion-controlled (Fickian behavior), and the diffusion is facilitated by electrostatic interactions. In contrast, at pH 5.5, drug release is influenced not only by diffusion but also by polymer chain relaxation and electrostatic interactions, resulting in a more complex, non-Fickian release mechanism. Furthermore, for DCF-C, DCF-F, and DCF-G, the porosity of the DC microparticles plays a critical role in drug release. As porosity decreases, the release profiles slow down, and the mechanism becomes more diffusion-controlled. In particular, DCF-G, which is expected to have the smallest pore size, exhibits a larger fraction of DOX release via Fickian diffusion at pH 5.5 due to stronger electrostatic repulsive forces. In contrast, at pH 7.4, the diffusion is more restricted by electrostatic attractions, leading to a slower, diffusion-controlled release.

### 2.4. Computational Study

All the density functional theory (DFT) simulations were performed using the Gaussian 16 program [52]. The geometry optimizations of DOX and DCFs A, C, and E of Table 1 were performed using a 6-31G(d) basis set and M062X functional [53]. The complexes formed from DOX and each of the DCFs were optimized at the M062X/3-21G level of theory, and the local minima of the optimized geometries were further confirmed with no imaginary frequencies using harmonic vibrational frequency analysis. Further, the corresponding complexation energies were obtained from single point calculation at the M062X/6-31G(d) level of theory. The selected hybrid meta-GGA-based Minnesota functional has been proven to be efficient at calculating the complexation energy [54]. The complexation energies obtained for DCFs A, C, and E with DOX were −114.28, −106.83, and −67.80 kJ/mole, respectively.

**Figure 6:**
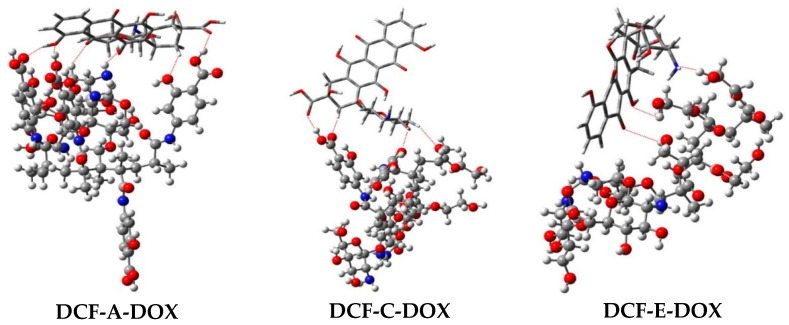
Optimized geometries of the complexes DCF-A-DOX, DCF-C-DOX, and DCF-E-DOX obtained at the M062X/6-31G(d)//M062X/3-21G level of theory. Hydrogen bonds are highlighted with red dotted lines. Doxorubicin is represented with the tube model, and the DCFs are represented with the ball and stick model.

**Figure 7:**
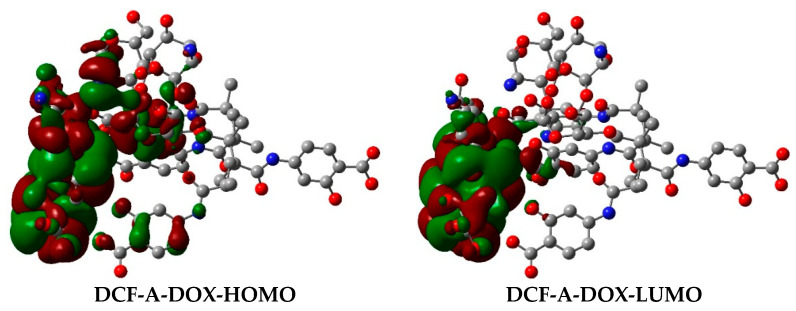
Charge transfer phenomenon in frontier molecular orbitals of the DCF-A-DOX complex. Hydrogen atoms were omitted for clarity.

As shown in Figure 6, the high complexation energies can be attributed to the intermolecular hydrogen bonds (H-bonds) and π–π interactions formed between DOX and DCFs. It is interesting to observe that the number of H-bonds formed in each complex varies: six for DCF-A-DOX, four for DCF-C-DOX, and three for DCF-E-DOX. All H-bonds observed in complexes DCF-A-DOX and DCF-C-DOX are those formed between hydrogen and oxygen atoms, whereas in the case of complex DCF-E-DOX, two H-bonds were formed between hydrogen and oxygen atoms, and the remaining H-bond was formed between hydrogen and nitrogen atoms. In addition, as shown in Figure 7, the strong interaction between DOX and DCF is the supporting evidence of the charge transfer phenomenon observed in the frontier molecular orbitals. From the theoretical simulation results, it can be confirmed that DCF-A is the most efficient, over the other two DCFs.

## 3. Materials and Methods

### 3.1. Materials

Medium molecular weight CTS, 4-amino salicylic acid (4-ASA), HEMA, methacrylic anhydride (MA), ceric ammonium nitrate (CAN), and DOX were procured from Sigma-Aldrich (Milwaukee, WI, USA). The NaNO_2_, NaOH, methanol, and acetic acid were analytical grade, which were purchased from Tokyo Chemical Industry (TCI) (Tokyo, Japan) and used without further purification.

### 3.2. Synthesis of Monomer

The 2-HMBA monomer was synthesized (Scheme S1) according to the previous literature [55] by selective amidation of 4-aminosalicylic acid (4-ASA) with methacrylic anhydride (MA) at 0 °C using dry acetone as a solvent. To 50 mL of 10% 4-ASA solution of dry acetone under constant stirring, 30 mL of acetone solution containing the required amount of MA was added dropwise under nitrogen atmosphere for 15 min and allowed to react for an additional 2 h with continuous stirring. After completion of reaction, the solvent was removed with a rotary evaporator to obtain a crude product. The remaining solid residue was washed several times with mixed solvent of methanol/water (4/1, *v*/*v*). The crystals were recrystallized from the mixed solvent of methanol/water (2/1. *v*/*v*) and dried under vacuum to obtain pure 2-HMBA (yield, 60%).

### 3.3. Depolymerization of Chitosan

Medium molecular weight CTS was depolymerized by a chemical oxidation reaction to achieve the desired molecular weights, according to the methods described by Mao et al. with a minor modification [56]. Briefly, five milliliters of NaNO_2_ solution (14.0 g/L) in deionized water was added to the solution of CTS (2 gms of CTS in 100 mL of 6% (*v*/*v*) acetic acid) at room temperature under stirring for 1 h. The reduced-molecular-weight CTS formed was precipitated by adjusting pH to 9.0 with 4 molar NaOH solution. The white-to-yellowish precipitate obtained was filtered and washed 2~3 times with acetone. The precipitate was again dissolved in a minimum volume of acetic acid (0.1 M) and purified by dialysis (Sigma dialysis tubes MW cutoff 12 kDa) against deionized water with periodic bath changes thrice a day. The dialyzed product was freeze-dried and stored at 4 °C.

### 3.4. Fabrication of Microparticles

**Scheme 1:**
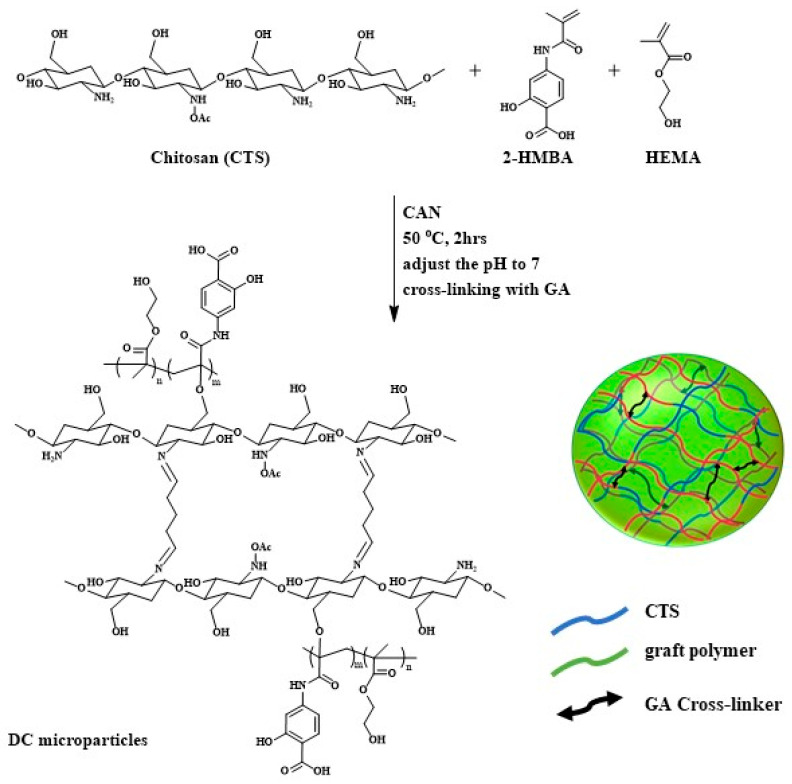
Schematic representation of fabrication of CTS/PHCH microparticles.

Microparticles with different drug carrier formulations (DCFs) were fabricated via redox-mediated graft copolymerization onto the linear chains of CTS, as illustrated in Scheme 1. The required amount of CTS was dissolved in 50 mL of double-distilled water and purged with nitrogen for 10 min. When the temperature reached 50 °C, the required amount of CAN was added for radical initiation on the CTS chains. Then, grafting on CTS was achieved by the addition of 10 mL of pre-dissolved monomer mixture of 2-HMBA and HEMA and continued to react for 2 h. After the polymerization was completed, the reaction solution was cooled to room temperature, and the pH of the solution was increased to 7 by adding dilute (0.5 N) sodium hydroxide solution. When the solution became opaque, a certain amount of glutaraldehyde solution was added to complete the formation of microparticles by cross-linking.

### 3.5. Characterizations

The DC microparticles were characterized by Fourier-transform infrared (FTIR) spectroscopy, differential scanning calorimetry (DSC), and electron microscopies such as SEM and TEM. The FTIR spectra of DC microparticles and their drug-loaded entities were recorded in an Infrared Spectrometer (JASCO spectrophotometer, JASCO Inc., Easton, MD, USA) in KBr discs. The morphology of the DC microparticles was evaluated by scanning electron microscopy (SEM) observations. The microparticles dispersed on carbon tapes were sputter-coated with gold prior to the acquisition of SEM images, using a scanning electron microscope (Carl Zeiss AG- Supra 25, Oberkochen, Germany). TEM analysis was performed with a transmission electron microscope (Hitachi H7600 TEM, Tokyo, Japan) after the deposition of samples on the copper grids covered with a perforated carbon film with the aid of water-dispersed samples. The TGA results were acquired using a thermal analyzer (Netzsch STA 449 F3 Jupiter, Forschungszentrum Jülich, Germany). The powdered samples (8~10 mgs) were crimped in an Al pan and heated at a 10 °C per minute temperature ramp over a temperature range of 35 to 650 °C under N_2_ atmosphere.

### 3.6. Drug Loading and Drug Entrapment Efficiency

The drug DOX was encapsulated into DC microparticles by stirring overnight with a DOX solution at a 50 μg mL^−1^ concentration. After being centrifuged, the microparticles were washed three times to remove free DOX and dried for further use. The supernatant DOX solution was assayed by the UV-Vis absorption signals at 485 nm to know the encapsulation efficiency (% EE). Then, the DC microparticles (5 mg) loaded with DOX were dispersed into 10 mL of distilled water and stirred continuously at 100 rpm in a vibrating incubator at 37 °C for about one day. Finally, the microparticle suspension was centrifuged, and the supernatant solution was assayed by the UV-Vis absorption signals at 485 nm, then the amount of DOX encapsulated into the microparticles and the % of drug loading (% DL) using the absorption intensity was calculated. The percentage of EE (%) and the DL (%) were calculated using the following equations:

(1) $$EE\;\left(\%\right)=\frac{Amount\;of\;DOX\;present\;in\;the\;microparticles}{Amount\;of\;DOX\;used\;for\;loading}\times 100$$

(2) $$DL\;\left(\%\right)=\frac{Amount\;of\;DOX\;present\;in\;the\;microparticles}{Amount\;of\;microparticles}\times 100$$

### 3.7. Drug Release and Model Fitting

The release of DOX from the pH-sensitive microparticles was studied through the bath method, with the aqueous dispersions of DOX-encapsulated microparticles in the buffer solutions [57]. Typically, 2 mg of DOX-encapsulated microparticles were dispersed in 2 mL of release medium and stirred continuously in a shaking incubator with a shaking speed of 50 rpm at 37 °C (±0.5 °C). At predetermined time intervals, the samples were centrifuged, and the supernatant solution was subjected to UV-Vis, and the same amount of fresh release medium was added to the withdrawn samples.

The cumulated release rates were calculated by using Equation (3):

(3) $$Cumulative\;drug\;release\;\left(\%\right)=\frac{Amount\;of\;DOX\;released\;}{Total\;amount\;of\;DOX\;in\;the\;microparticles}\times 100$$

Each experiment was conducted in triplicate, and the data collected in this study were expressed as the mean value ± standard deviation (SD).

Then, the drug release data was fitted with different mathematical empirical models, like Higuchi’s square root of time equation, first-order, and zero-order, and semi-empirical models like the power law equation of Ritger–Peppas and weighted sum of non-linear time function of Peppas–Sahlin were fitted as follows:

Higuchi model:

(4) Qt= kH t12

First-order:

(5) $$Q_{t}=\;Q_{e}(1-e^{-k_{1}t})$$

Zero-order:

(6) $$Q_{t}=\;Q_{0}-\;k_{0}\;t$$

Ritger–Peppas:

(7) $$Q_{t}=\;\frac{M_{t}}{M_{\infty }}=\;k_{RP}\;t^{n}$$

Peppas–Sahlin:

(8) $$Q_{t}=\;\frac{M_{t}}{M_{\infty }}=\;k_{1}\;t^{0.45}+\;k_{2}\;t^{0.9}$$

where Q_0_ is initial amount of drug present in the microparticles; Q_t_ is percentage of drug released at time t; and k_0_, k_1_, k_H_, and k_C_ are the rate constants of the respective equations. Here the empirical models describe the information about the types of drug release, while the semi-empirical Ritger–Peppas model derives the actual mechanism of drug release, i.e., whether the drug release belongs to Fickian, Non-Fickian, or case II (anomalous) transport. However, the Peppas–Sahlin models gives the fraction of drug release due to the diffusion at a given instant of time t using the following equation:

(9) F=[1+k2k1t0.45 ]−1

### 3.8. Computational Study

The calculations of density functional theory with a hybrid functional B3LYP (Becke’s three-parameter hybrid functional) used Gaussian 16 software. The optimized molecular structures of DOX and DCFs (4 repeating units of polymer and CTS rings) and corresponding vibrational assignments of DOX and DC microparticles were investigated with a 6-31G(d) basis set and M062X functional.

## 4. Conclusions

This work has described the design, synthesis, physicochemical characterization, and drug release evaluation of chitosan-based DC microparticles. By varying the comonomer (2-HMBA, HEMA) ratio, different DCFs of DC microparticles were produced that exhibited a wide range of physicochemical and drug release properties. FTIR and TGA studies confirm the uniform distribution of DOX in the DC microparticles, and DFT calculations indicate that the DOX molecules can form hydrogen bond interactions with DC microparticles. All the DCFs have optimal drug loading capacity, and the DOX release profiles are pH-dependent and controlled in manner. The higher loading capacity and faster in vitro DOX release properties of microparticles at pH 5.5 were due to the large number of polar groups present in the microparticles. Further, the same was confirmed from computational studies. The experimental results of the drug cumulative release were as expected according to the extent of protonation predicted by the Ritger–Peppas equation. Finally, this data suggests that the prepared microparticles are suitable to release DOX at cancer tissue to maximize the drug’s efficacy and reduce the side effects.

## Figures and Tables

**Table 1:** DCF microparticle feeding ratio of monomers and encapsulation efficiency and drug loading.

	CTS (mg)	HEMA (mg)	2-HMBA (mg)	CAN (mg)	GA (mmol)	EE (%)	DL (%)
DCF-A	50	0	100	10	0.02	39.91 ± 1.2	91.77 ± 0.9
DCF-B	50	25	75	10	0.02	37.96 ± 1.1	88.5 ± 1.3
DCF-C	50	50	50	10	0.02	36.82 ± 0.6	85.87 ± 1.7
DCF-D	50	75	25	10	0.02	35.55 ± 1.8	80.25 ± 1.1
DCF-E	50	100	0	10	0.02	34.83 ± 1.4	77.86 ± 1.6
DCF-F	50	50	50	10	0.0125	37.59 ± 1.1	88.26 ± 1.3
DCF-G	50	50	50	10	0.05	30.25 ± 1.6	54.51 ± 2.5

**Table 2:** Empirical kinetic model fitting parameters at pH 5.5 and 7.4.

			DCF-A	DCF-B	DCF-C	DCF-D	DCF-E	DCF-F	DCF-G
pH 5.5	Higuchi	r^2^	0.8110	0.8650	0.8402	0.8632	0.8639	0.7741	0.9625
		k_H_	0.2639	0.2671	0.2566	0.2245	0.2784	0.2665	0.2937
		SSR	0.1487	0.1028	0.1152	0.0737	0.1127	0.1886	0.0315
	0 Order	r^2^	0.5312	0.6058	0.5664	0.6212	0.6034	0.4867	0.7973
		k_0_	0.0597	0.0620	0.0587	0.0527	0.0646	0.0594	0.0731
		Q_0_	0.4480	0.3907	0.4308	0.3704	0.3981	0.4675	0.2466
		SSR	0.3687	0.3001	0.3126	0.2042	0.3284	0.4285	0.1699
	First Order	r^2^	0.9840	0.9851	0.9725	0.9219	0.9898	0.9922	0.9924
		k_1_	0.8897	0.7191	0.8513	0.9431	0.6738	0.9173	0.3498
		Q_e_	0.9270	0.9013	0.9021	0.7727	0.9398	0.9480	0.9408
		SSR	0.0126	0.0113	0.0198	0.0421	0.0084	0.0065	0.0064
pH 7.4	Higuchi	r^2^	0.8428	0.7791	0.8206	0.8143	0.8533	0.8000	0.7744
		k_H_	0.0385	0.0439	0.0500	0.0550	0.0670	0.0313	0.0932
		SSR	0.0203	0.0400	0.0402	0.0506	0.0568	0.0180	0.1850
	0 Order	r^2^	0.6272	0.5505	0.6000	0.5871	0.6357	0.5743	0.5515
		k_0_	0.0045	0.0051	0.0059	0.0064	0.0079	0.0036	0.0108
		Q_0_	0.1414	0.2052	0.2166	0.2251	0.2554	0.1377	0.4585
		SSR	0.0482	0.0813	0.0896	0.1124	0.1410	0.0383	0.3678
	First Order	r^2^	0.9234	0.9235	0.9088	0.9380	0.9177	0.9232	0.9095
		k_1_	0.3569	0.5422	0.4724	0.4161	0.3610	0.4862	0.6154
		Q_e_	0.2810	0.3555	0.3911	0.4206	0.4983	0.2466	0.7722
		SSR	0.0099	0.0138	0.0204	0.0169	0.0319	0.0069	0.0742

**Table 3:** Semi-empirical kinetic model fitting parameters at pH 5.5 and 7.4.

			DCF-A	DCF-B	DCF-C	DCF-D	DCF-E	DCF-F	DCF-G
pH 5.5	Ritger–Peppas	n	0.2393	0.2790	0.2412	0.2418	0.2859	0.2320	0.4330
		kH	0.5782	0.5106	0.5587	0.4872	0.5200	0.5994	0.3481
		r^2^	0.9548	0.9609	0.9780	0.9880	0.9540	0.9306	0.9707
		SSR	0.0356	0.0297	0.0159	0.0065	0.0381	0.0579	0.0246
	Peppas–Sahlin	r^2^	0.9858	0.9859	0.9972	0.9736	0.9833	0.9752	0.9733
		k1	0.6806	0.5937	0.6480	0.5430	0.6084	0.7166	0.3715
		k2	−0.1190	−0.0916	−0.1104	−0.0867	−0.0929	−0.1303	−0.0143
		SSR	0.0111	0.0108	0.0020	0.0142	0.0138	0.0207	0.0224
	Fraction of DOX released by Fickian diffusion at 4 h.		0.7540	0.7765	0.7588	0.7704	0.7782	0.7467	0.9330
pH 7.4	Ritger–Peppas	n	0.2392	0.1915	0.2055	0.2176	0.2315	0.2032	0.1807
		kH	0.1299	0.1957	0.2049	0.2099	0.2363	0.1302	0.4432
		r^2^	0.9601	0.9648	0.9779	0.9618	0.9767	0.9657	0.9744
		SSR	0.0052	0.0064	0.0050	0.0104	0.0090	0.0031	0.0210
	Peppas–Sahlin	r^2^	0.9740	0.9511	0.9623	0.9751	0.9834	0.9604	0.9286
		k1	0.1176	0.1678	0.1766	0.1867	0.2108	0.1130	0.3706
		k2	−0.0114	−0.0181	−0.0182	−0.0192	−0.0206	−0.0119	−0.0402
		SSR	0.0034	0.0089	0.0084	0.0068	0.0064	0.0036	0.0586
	Fraction of DOX released by Fickian diffusion at 4 h.		0.8468	0.8324	0.8387	0.8390	0.8458	0.8358	0.8317

## Data Availability

The raw data supporting the conclusions of this article will be made available by the authors on request.

## Supplementary Materials

The following supporting information can be downloaded at: https://www.mdpi.com/article/10.3390/ijms262110460/s1.
