# Supplementary material for: Fabrication and Computational Study of pH-Responsive Chitosan/Poly(HEMA-co-2-HMBA) Microparticles for Controlled, Site-Specific Doxorubicin Delivery
Source: Int J Mol Sci. 2025 Oct 28;26(21):10460. doi: 10.3390/ijms262110460 (PMC12607976; doi:10.3390/ijms262110460)
Supplement: Supplementary file 1 [file ijms-26-10460-s001.zip › ijms-3916258-supplementary.pdf]

Article

Fabrication and Computational Study of pH-Responsive Chitosan/Poly(HEMA-co-2-HMBA) Microparticles for Controlled, Site-Specific Doxorubicin Delivery

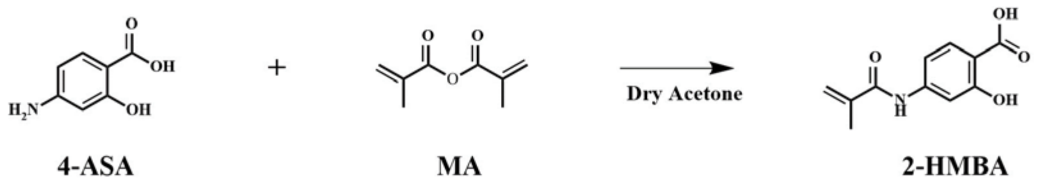

Scheme S1. Schematic representation for the synthesis of 2-HMBA.

Table S1. TGA data of DOX, DC microparticles and their DOX loaded microparticles

Material	Weight loss stages (°C)	Weight loss (%)	Weight loss assignment
**DC**	156 ~ 384	54.70	Thermal decomposition of polymer network of DC micro gels.
**DOX.HCl**	147 ~ 257	27.50	Thermal degradation of DOX.
	257 ~ 364	12.79	Thermal decomposition of DOX degradants.
**DOX loaded DC**	156 ~ 384	45.55	Thermal decomposition of polymer network including DOX.
